# Islet Brain 1 Protects Insulin Producing Cells against Lipotoxicity

**DOI:** 10.1155/2016/9158562

**Published:** 2015-11-09

**Authors:** Saška Brajkovic, Mourad Ferdaoussi, Valérie Pawlowski, Hélène Ezanno, Valérie Plaisance, Erik Zmuda, Tsonwin Hai, Jean-Sébastien Annicotte, Gérard Waeber, Amar Abderrahmani

**Affiliations:** ^1^Service of Internal Medicine, Centre Hospitalier Universitaire Vaudois and University of Lausanne, 1011 Lausanne, Switzerland; ^2^University of Lille, European Genomic Institute for Diabetes (EGID) FR 3508, UMR CNRS 8199, Faculty of Medicine West, 1 place de Verdun, 59045 Lille, France; ^3^Department of Pharmacology and the Alberta Diabetes Institute, University of Alberta, Edmonton, AB, Canada; ^4^University of Lille, EGID FR 3508, Department of Endocrine Surgery, Lille University Hospital, UMR INSERM 1190, Lille, France; ^5^Department of Molecular and Cellular Biochemistry, Ohio State University, 1060 Carmack Road, Columbus, OH, USA

## Abstract

Chronic intake of saturated free fatty acids is associated with diabetes and may contribute to the impairment of functional beta cell mass. Mitogen activated protein kinase 8 interacting protein 1 also called islet brain 1 (IB1) is a candidate gene for diabetes that is required for beta cell survival and glucose-induced insulin secretion (GSIS). In this study we investigated whether IB1 expression is required for preserving beta cell survival and function in response to palmitate. Chronic exposure of MIN6 and isolated rat islets cells to palmitate led to reduction of the IB1 mRNA and protein content. Diminution of IB1 mRNA and protein level relied on the inducible cAMP early repressor activity and proteasome-mediated degradation, respectively. Suppression of IB1 level mimicked the harmful effects of palmitate on the beta cell survival and GSIS. Conversely, ectopic expression of IB1 counteracted the deleterious effects of palmitate on the beta cell survival and insulin secretion. These findings highlight the importance in preserving the IB1 content for protecting beta cell against lipotoxicity in diabetes.

## 1. Introduction

Type 2 diabetes is one of the major health challenges of the 21st century. The disease arises when beta cells produce insufficient insulin to meet the increased hormone demand, caused by insulin resistance or growth of tissues such as liver, muscle, and adipose tissues. Although genome-wide association studies revealed a genetic contribution in the etiology of the disease [1], the environmental risks factors are very likely the most prominent cause of beta cell decline in the vast majority of cases [2]. Lifestyle changes such as lack of physical activity together with excessive adiposity contribute to chronic elevation of the circulating plasma saturated free fatty acids (FFAs). Numerous studies have highlighted that chronic exposure to elevated levels of FFAs, in particular palmitate, is detrimental by promoting insulin resistance and beta cell dysfunction [3]. The beta cell failure elicited by palmitate includes a defect in their secretory capacity to respond to glucose and a loss of beta cell mass by apoptosis [4–8]. These diabetogenic effects of palmitate are in part achieved by modulating the expression and activity of proapoptotic and antiapoptotic proteins [3, 9–20].

The mitogen activated protein kinase 8 interacting protein 1, also named islet brain 1 (IB1) or c-Jun N Terminal Kinase- (JNK-) interacting protein 1 (JIP1), is mainly expressed in islet beta cells and is one of the key antiapoptotic factors of this cell type [21–24]. Reduction of the IB1 content in insulin producing and islets cell increases apoptosis [25–27]. A wealth of data reports the diminution of IB1 level, as a major mechanism through which inflammatory cytokines cause beta cell apoptosis [22, 23, 25–29]. Some studies have ascribed the protective role of IB1 to the regulation of JNK pathway, although the exact mechanism of this regulation is still unclear [30, 31]. Reduction of IB1 expression may activate phosphorylation of JNK targets [30]. A mutation within the coding region of this gene has been associated with a rare and monogenic form of diabetes and induces beta cell death* in vitro* [23].

Conversely, overexpression of IB1 renders cells more resistant to apoptosis induced by cytokines [22, 23, 26, 27, 29]. Moreover, induction of IB1 is a major target of the glucagon-like peptide 1 mimetics for preventing beta cell death [26]. However, the role of IB1 in the context of lipotoxicity has not been reported thus far. In this report, we demonstrated the roles of IB1 in palmitate-induced beta cell death and function and described the regulation of IB1 by palmitate at both the transcriptional and posttranslational levels.

## 2. Material and Methods

### 2.1. Materials

Palmitate (sodium salts) was obtained from Sigma-Aldrich (St. Louis, MO). The saturated fatty acid was coupled to bovine serum albumin by 1 h agitation at 37°C and freshly prepared for each experiment [32]. This procedure yielded BSA-coupled fatty acids in a molar ratio of 5 : 1. The MG132 compound was purchased from Sigma-Aldrich (St. Louis, MO). The antibodies against IB1, mSIN3, and c/ebp*β* were obtained from Santa Cruz Biotechnology (CA, USA).

### 2.2. Islets Preparation, Cell Culture, and Transfection

Rat islets were isolated from the pancreas of Sprague-Dawley rats (male, at body weight of 250–350 g) by ductal injection of collagenase. The purification and culture of islets were conducted as described [29]. The mouse insulin-secreting cell line MIN6 was cultured in DMEM glutamax medium (Invitrogen, Carlsbad, CA) supplemented with 15% FCS, 50 U/mL penicillin, 50 *μ*g/mL streptomycin, and 70 *μ*M *β*-mercaptoethanol [33]. The rat insulin-secreting cell line INS-1E was maintained in RPMI 1640 medium supplemented with 10% FCS, 1 mM Sodium Pyruvate, 50 *μ*M *β*-mercaptoethanol, and 10 mM Hepes [26]. The plasmid encoding HA-IB1-WT and siRNA duplexes directed against IB1 (si-IB1), GFP (si-GFP), or ICER (siICER) were previously described [26, 34]. Plasmids or the siRNA duplexes were introduced using the Lipofectamine 2000 (Invitrogen AG) exactly as described [26].

### 2.3. Measurement of Insulin Secretion

The MIN6 cells (10^5^) were plated in 24-well dishes. Two days after transfection, cells were washed twice with PBS. Thereafter, cells were preincubated in KRBH buffer (140 mM NaCl, 3.6 mM KCl, 0.5 mM NaH_2_PO_4_, 0.5 mM MgSO_4_, 1.5 mM CaCl_2_, 2 mM NaHCO_3_, 10 mM HEPES, 0.1% bovine serum albumin, and pH 7.4) containing 2 mM glucose for 1 hour. Afterward medium was changed with KRBH buffer containing 2 mM glucose corresponding to basal state or with 20 mM glucose for an additional 45 minutes. Insulin secretion was measured by EIA (SPI-BIO) according to manufactured protocol.

### 2.4. Western Blotting

The cells were scrapped in the PBS and lysed by using a NP-40 lysis buffer (50 mM Tris-HCl, pH 8, 150 mM NaCl, and 1% NP-40) supplemented with antiproteases and antiphosphatases (Roche). 25–40 *μ*g of total protein extracts was separated on 10% SDS-polyacrylamide gel and electrically blotted to nitrocellulose membrane. The proteins were detected using a buffer containing 0.1% Tween 20 and 5% milk and incubated overnight at 4°C with specific primary antibodies and were visualized with IRDye 800 or IRDye700 (Rockland) as secondary antibodies. Quantification was realized using the Odyssey Infrared Imaging System (Li-COR).

### 2.5. Reverse Transcription Coupled with Quantitative PCR (RT-qPCR)

Total RNA was extracted using guanidium thiocyanate-phenol-chloroform RNA purification method. Reverse transcription was performed as described [34]. Real-time quantitative-PCR assays were carried out on the Bio-Rad MyiQ Real-Time PCR Detection System using iQ SyBr Green Supermix (Bio-Rad) as the amplification system with 100 nM primers and 2 *μ*L of template (RT product) in 20 *μ*L of PCR volume and annealing temperature of 59°C. Primers sequences were as follows: mouse* Ib1*, sense 5′-ACA AGG GCA ATG ATG TCC TC-3′ and antisense 5′-TTT ATT TCC CTT GGC CTC C-3′; mouse housekeeping ribosomal protein, large P0 (*Rplp0*), sense 5′-ACCTCCTTCTTCCAGGCTTT-3′ and antisense 5′-CCACCTTGTCTCCAGTCTTT-3′; mouse* Bcl2*, sense 5′-CTCCCGATTCATTGCAAGTT-3′ and antisense 5′-TCTACTTCCTCCGCAATGCT-3′.

## 3. Results

### 3.1. Reduction of* Ib1* Content in MIN6 Cells by Palmitate Relied on the Transcriptional Repressor ICER and Proteasome-Mediated Degradation

**Figure 1:**
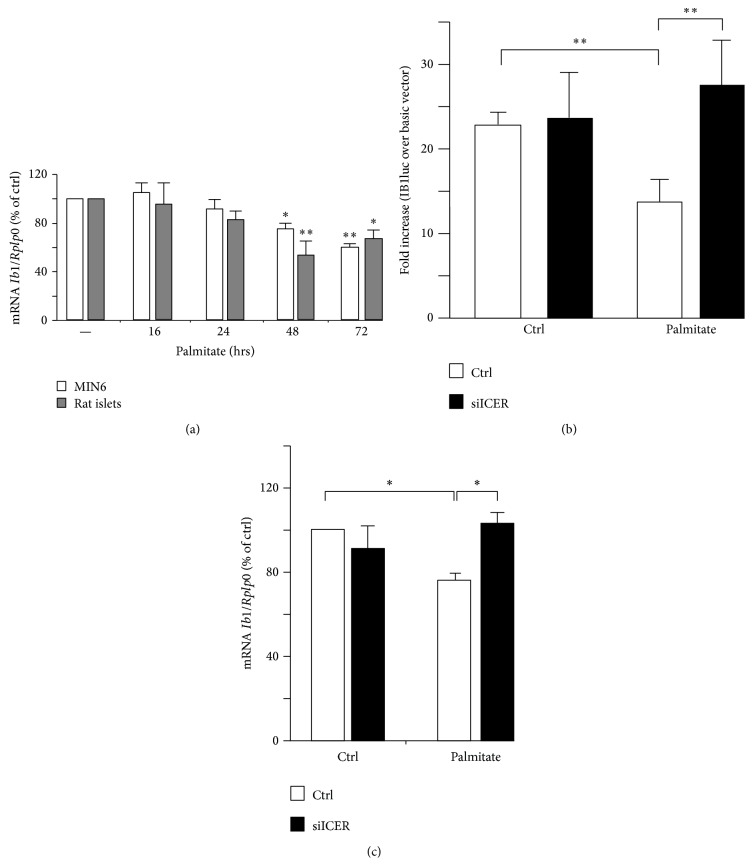
Effect of palmitate on the* Ib1* mRNA level. (a) Quantification of* Ib1* mRNA level by RT-qPCR from MIN6 cells (*open bar*) and isolated rat islets (grey bar) cultured with 0.5 mM palmitate or BSA (−) at different indicated times. (b) Assessment of Ib1 transcriptional activity in MIN6 cells cultured with palmitate. Cells were transiently transfected with a luciferase reporter construct driven by the 731 bp fragment of the human* MAPK8IP1* promoter (IB1luc). Palmitate was added to the medium 24 hrs after transfection and luciferase activity was measured 48 hrs later. To test the role of ICER, IB1luc was cotransfected together with duplexes of control small interfering RNA (siGFP,* open bar*) or siRNA directed specifically against ICER (siICER,* filled bar*). The data are expressed as fold increase over the control vector pGL3basic and are the mean ± SEM of three independent experiments. (c) Role of ICER in the drop of* Ib1* mRNA induced by palmitate. The* Ib1* mRNA was measured by RT-qPCR in MIN6 cells that were transfected with duplexes of either siGFP (*open bar*) or siICER (*filled bar*). After transfection, (24 hrs) the cells were cultured with BSA (ctrl) or 0.5 mM palmitate for additional 48 hrs. The results were normalized against* Rplp0* and the expression levels from cells cultured with BSA were set to 100%. Data are the mean ± SEM of 3 independent experiments (^*∗∗*^ *P* < 0.01; ^*∗*^ *P* < 0.05).

**Figure 2:**
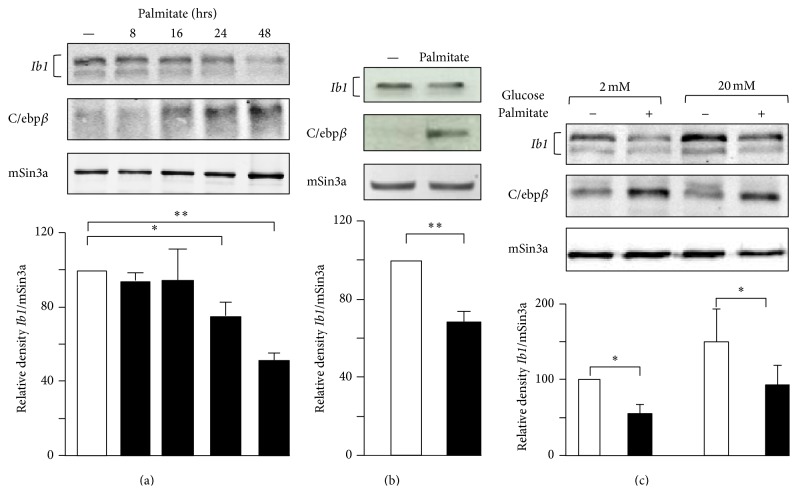
Effect of palmitate on the Ib1 protein level. Total protein was prepared from (a) MIN6 cells or (b) isolated rat islets that were cultured with 0.5 mM palmitate or BSA (−) at different indicated incubation times and for 24 hrs, respectively or (b) incubated with BSA or palmitate and (c) INS-1E exposed to BSA (−) or 0.5 mM palmitate in the presence of 2 mM or 20 mM glucose. As a positive control for the palmitate efficacy, the expression of C/ebp*β* was monitored. Immunoblotting of mSin3a was done as negative control. The* Graphs* below the blots depict the densitometric analysis. The sum of the Ib1 band intensities of cells treated with BSA was set at 100%. The figure shows the results of a representative experiment out of five. Data are the mean of ± SEM of 3 independent experiments (^*∗∗*^ *P* < 0.01; ^*∗*^ *P* < 0.05).

**Figure 3:**
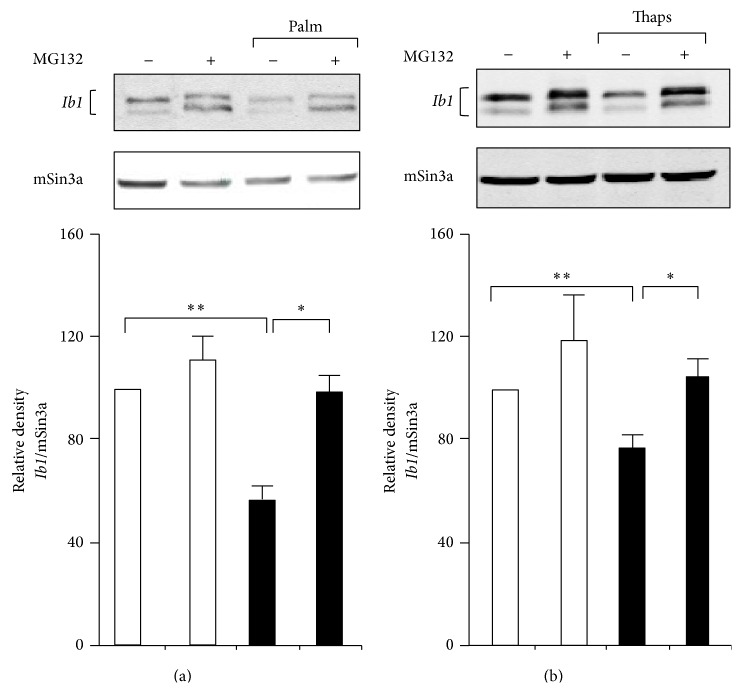
Effect of the proteasome inhibitor MG132 on the decrease of Ib1 content caused by palmitate. Ib1 content was measured from MIN6 cells exposed to (a) 0.5 mM palmitate or (b) 1 *μ*M thapsigargin (thaps) for 48 hrs in the presence or absence of 1 *μ*M of the proteasome inhibitor MG132. The Ib1 level was normalized against the mSin3a. The figure shows the result of a representative experiment out of three. The results are expressed as the mean ± SEM of three independent experiments (^*∗∗*^ *P* < 0.01; ^*∗*^ *P* < 0.05).

A large number of reports have confirmed the adverse effects of palmitate on function and survival of isolated islets and different insulin-secreting cells including MIN6 cells [11, 13, 15, 19]. For this reason we chose to monitor the* Ib1* mRNA level in MIN6 cells and isolated rat islets that were cultured with palmitate. RT-qPCR showed reduction of* Ib1* mRNA in islet and MIN6 cells cultured with palmitate for 48 and 72 hrs (Figure 1(a)). Because palmitate modulates the activity of several transcription factors [11], we tested the hypothesis that the decreased* Ib1* mRNA levels resulted from reduced transcriptional activity of its promoter. The human proximal* IB1* promoter contains several key elements that promote expression and regulation of the gene in beta cell [35]. A 731 bp fragment of the proximal promoter has been cloned upstream of the luciferase reporter (*IB1*luc) and is highly active in insulin producing cells [24]. As previously observed, luciferase activity of the IB1-luc construct was 20–25-fold higher than the promoterless control vector in MIN6 cells (Figure 1(b)). This activity was reduced by twofold when the cells were cultured in the presence of palmitate (Figure 1(b)). The* IB1* promoter contains a cAMP response element (CRE) [26]. This element binds the inducible cAMP early repressor (ICER) [26], an antagonist of the CRE-binding protein (CREB). ICER expression rises up in beta cells incubated with palmitate [36]. We have previously demonstrated that overexpression of ICER represses the promoter activity of* IB1*-luc in beta cells [26]. To investigate whether ICER links palmitate to reduced Ib1 mRNA levels, we transfected MIN6 cells with siRNA directed against ICER (siICER) that we previously validated in beta cells [28, 34, 37]. Interestingly, silencing of ICER restored* IB1*luc activity and* Ib1* mRNA levels in the presence of palmitate (Figures 1(b) and 1(c)), supporting a role for ICER in the reduction of* Ib1* expression induced by palmitate. Activating transcription factor 3 (ATF3), which also binds to the CRE site, is a potent repressor of gene expression induced by palmitate in beta cells [35, 38, 39]. However, the Ib1 expression was neither reduced in cells in which Atf3 was overexpressed (see supplementary Figure 1a in Supplementary Material available online at http://dx.doi.org/10.1155/2016/9158562) nor increased in islets cells from atf3 knockout mice, thus ruling out a role for Atf3 in the loss of* Ib1* mRNA caused by the saturated fatty acid (supplementary Figure 1b). Two Ib1 isoforms, one corresponding to the full length protein and one from the use of an alternative promoter [26], were detected in MIN6 cells by immunoblotting experiments (Figure 2(a)) [26, 27]. A significant reduction in Ib1 protein levels was apparent after 24 hrs treatment of cells with palmitate (Figure 2(a)). These results were confirmed in isolated rat islets cultured with palmitate for 24 hrs (Figure 2(b)). Palmitate hampers insulin expression, secretion, and cell survival by inducing the expression of C/EBP*β* [11]. Interestingly we observed that the decreased Ib1 protein level was concomitant with the increased C/EBP*β* protein levels (Figures 2(a) and 2(b)). Chronic hyperglycemia potentiates the harmful effects of palmitate [9] in INS-1E cells but not in MIN6 or isolated human islets [40]. To determine whether the effects of palmitate were potentiated by glucose, Ib1 protein levels were quantified in INS-1E cells cultured with palmitate in the presence of low or high glucose concentration (5 or 20 mmol/L glucose, resp.). A similar reduction of Ib1 by palmitate was observed upon low or high glucose concentration (Figure 2(c)), indicating that palmitate decreases the expression of high glucose concentration. Since decreased Ib1 protein levels occurred prior to the decrease of its mRNA levels (Figures 1(a) and 2(a)), this suggests that the reduction of* Ib1* mRNA is not the only mechanism affecting its protein content. The fatty acid affects beta cell survival and function through ER stress dependent pathways [3, 15–17]. Palmitate impairs Ca^2+^ influx to ER by affecting sarcoendoplasmic-reticulum pump Ca^2+^-ATPase (SERCA), also known as ATP2A2 activity [19]. Defective cytosolic Ca^2+^ leads to proteasome-mediated degradation [41]. To test the hypothesis that the loss of Ib1 content involves proteasome, MIN6 cells were coincubated with palmitate and the proteasome inhibitor MG132. Treatment of cells with this chemical compound efficiently restored Ib1 protein levels in the presence of palmitate (Figure 3(a)). In addition, thapsigargin (thaps), an ER stress inducer that promotes Ca^2+^-induced degradation evoked by proteasome [42], reduced Ib1 protein content in MIN6 and INS-1E cells (Figure 3(b)).Under these experimental conditions, MG132 treatment efficiently restored Ib1 protein level in the presence of thaps (Figure 3(b)).

### 3.2. Overexpression of* Ib1* Counteracts the Deleterious Effects of Palmitate on Glucose-Induced Insulin Secretion and Cell Survival

**Figure 4:**
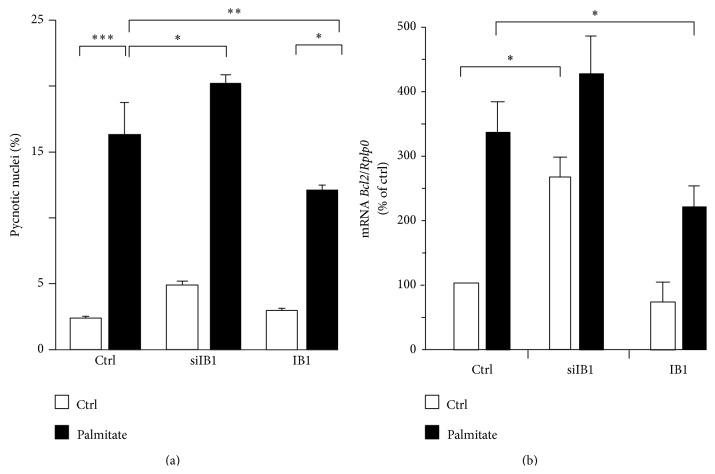
Role of Ib1 in apoptosis induced by palmitate. (a) MIN6 cells were transfected with the siRNA duplex directed against IB1 (siIB1) or control siRNA (siGFP, Ctrl) or the plasmids coding for the wild type HA-tagged IB1 (IB1). For scoring death, 0.5 mM palmitate (*filled bars*) or BSA (Ctrl,* open bars*) was added to the cells medium 24 hrs after transfection. The rate of apoptosis was scored by counting pycnotic nuclei in cells exposed for 48 hrs to palmitate. Results are expressed as mean ± SEM of 3 independent experiments (^*∗*^ *P* < 0.05; ^*∗∗*^ *P* < 0.01; ^*∗∗∗*^ *P* < 0.001). (b) For the quantification of* Bcl2*, total RNA from transfected cells with siGFP, siIB1, and IB1 was prepared and subjected to qPCR. The levels of* Bcl2* were compared in cells incubated with BSA (*open bars*) and 0.5 mM palmitate (*filled bars*) for 48 hrs. The mRNA were normalised against* Rplp0* and those of the control cells were set to 100%. Data are the mean ± SEM of five independent experiments (^*∗*^ *P* < 0.05).

**Figure 5:**
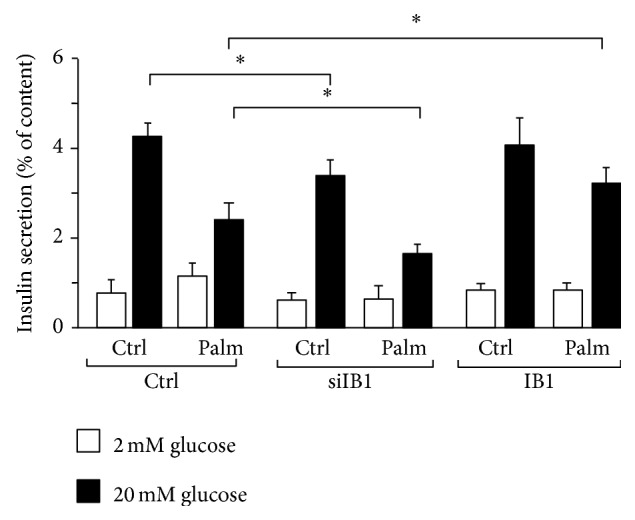
Role of Ib1 in impaired glucose-induced insulin secretion caused by palmitate. For measurement of insulin secretion, MIN6 cells were transiently transfected with the control siRNA (*open bar*) or siIB1 (*filled bar*) or the plasmids coding for the wild type HA-tagged IB1 (IB1). 48 hrs after transfection, insulin secretion was stimulated by preincubating the cells for 30 min in Krebs-Ringer buffer containing 2 mmol/L glucose and, thereafter, incubating the cells with glucose 20 mmol/L. The amounts of insulin release and cellular contents during the incubation period were measured by EIA. The results are expressed as the ratio between the amounts of insulin released over the content and are the mean ± SEM of three independent experiments.

IB1 is required for glucose-induced insulin secretion and cell survival [23, 26, 43]. We investigated whether the decreased Ib1 level contributes to palmitate-induced cell death by ectopically expressing Ib1. As shown in Figures 4(a) and 4(b), IB1 partially rescued the cells as evidenced by the reduction in apoptotic cell number and increase in the mRNA level of* Bcl2*, an antiapoptotic gene. Conversely, silencing of Ib1 using a previously validated siRNA [26] potentiated the effect of palmitate on cell death (Figure 4(a)) with a concomitant increase of the* Bcl2* mRNA (Figure 4(b)). We next investigated whether the reduction of Ib1 by palmitate could contribute to defective glucose-induced insulin secretion. As previously shown [43], silencing of Ib1 in Min6 cells reduced glucose-induced insulin secretion (Figure 5), which was exacerbated in the presence of palmitate (Figure 5). Transient ectopic expression of Ib1 can overcome proteasome-mediated degradation of Ib1 elicited by cytokines [22, 23, 27, 44]. It has been previously shown that Ib1 overexpressing beta cells are more resistant to apoptosis [22, 23, 27, 44]. Interestingly and in line with these observations, Ib1 overexpressing Min6 cells improved their glucose-induced insulin secretion when chronically exposed to palmitate (Figure 5).

## 4. Discussion

Evidence for the potential diabetogenic role of palmitate by afflicting beta cell function and survival has been provided by a plethora of data from* in vitro* and* in vivo* experiments [3, 7, 12, 41]. Palmitate decreases beta cell survival by promoting apoptosis [19]. Reduction of the antiapoptotic IB1 expression is a major mechanism eliciting beta cell apoptosis in response to cytokines and oxidized LDL [25, 26, 28, 29]. However, its role in lipotoxicity has not been reported. In this report, we show that palmitate decreases Ib1 gene expression at both the transcriptional and posttranslational levels. At the transcriptional level, the effect is dependent on the transcriptional repressor ICER, since silencing of ICER dampened the ability of palmitate to reduce* Ib1* mRNA. Although the level of ATF3, another transcriptional repressor, is increased by palmitate [39], our data indicate that ATF3 is not necessary for palmitate to repress Ib1 gene expression. Interestingly, our data showed that diminution of IB1 protein content occurs earlier than the drop of* Ib1* mRNA and this was via a proteasome-mediated pathway. Overexpression of Ib1 protects beta cell against apoptosis triggered by cytokines [22, 25]. In line with this protective effect we observed that ectopic expression of Ib1 prevented the deleterious effect of palmitate on cell survival.

Beside its antiapoptotic role, Ib1 regulates glucose-induced insulin secretion [21, 23, 43]. Consistent with this metabolic function, inactivation of Ib1 alters insulin secretion stimulated by glucose [43]. Herein we confirmed that silencing of Ib1 mimics the effect of palmitate on insulin secretion. Moreover, ectopic expression of Ib1 partially restored glucose-induced insulin secretion in response to palmitate, indicating that exogenous expression of Ib1 compensates for the decrease of Ib1 content caused by palmitate. Ib1 is described as a scaffold protein that assembles the kinases involved in the JNK activation; however, paradoxically its function is to inhibit JNK activity [22, 25, 26, 29, 31]. JNK activation often (but not always) precedes JNK activity. The c-Jun transcription factor is a JNK target that is deemed to couple JNK activation to apoptosis [45]. JNK phosphorylates c-Jun and this could lead to apoptosis [45, 46]. Independent studies have shown that Ib1 level may be required for inhibiting phosphorylation of c-Jun [25, 27]. Overexpression of Ib1 level reduces phosphorylation of c-Jun caused by cytokines in islets and insulin producing cells [25, 27].

JNK pathway is activated in response to several diabetogenic stresses including oxidized LDL and cytokines [26, 29]. Increased JNK activity is a key mechanism coupling palmitate to beta cell dysfunction and ultimately cell death [3, 19], and inhibition of JNK activity alleviates the adverse effects of palmitate [3, 19]. Thus, our finding that palmitate reduces Ib1 expression may provide a potential mechanism for palmitate to increase JNK activity. There are three JNK isoforms identified so far [47]. All of them are present in beta cells [48]. There are growing studies pointing to divergent roles in JNK isoforms in beta cells. While JNK2 seems to be proapoptotic, JNK1 and JNK3 are antiapoptotic [48–50]. Therefore, further analyses are required to determine whether and how Ib1 may regulate each of the JNK isoforms. Understanding such regulation will permit us to elucidate the mechanism through which IB1 preserves beta cell against the harmful effects caused by palmitate. Inhibition of the JNK pathway has been proposed as a potential therapeutic way for treating beta cell failure in type 2 diabetes and some efforts are currently maintained to identify novel JNK inhibitors [51]. Future investigation of IB1 activity may help in finding out novel targets exploitable in the design of next innovative therapies of T2D.

## Supplementary Material

Supplementary Figure 1: Role of Atf3 on the Ib1 protein and mRNA levels.
