# Supplementary material for: Islet Brain 1 Protects Insulin Producing Cells against Lipotoxicity
Source: J Diabetes Res. 2015 Nov 9;2016:9158562. doi: 10.1155/2016/9158562 (PMC4655268; doi:10.1155/2016/9158562)

Supplementary Fig. 1. **a**) Quantification of Ib1 by Western Blotting in cells in which the Atf3 level was over-produced by transient transfection of the plasmid coding for Aft3 (pAtf3). The bars of the graph below correspond to the quantification of the *Ib1* mRNA by quantitative PCR in MIN6 cells transfected with Atf3 or the empty vector (Ctrl). The mRNA levels were normalized against the *Rplp0* and the expression levels from wild type islets were set to 100%. Data are the mean of ± SEM of 3 independent experiments. **b**) Measurement of Ib1 content in islets of Atf3 knockout mice. Quantification of Ib1 by Western Blotting in isolated islet cells from wild type littermates and Atf3 knockout mice ($Atf3^{-/-}$) that were incubated with thapsigargin (Thaps) for the indicated times. The bars of the graph below correspond to the quantification of the *Ib1* mRNA by qRT-PCR. The mRNA levels were normalized against the *Rplp0* and the expression levels from wild type islets were set to 100%.

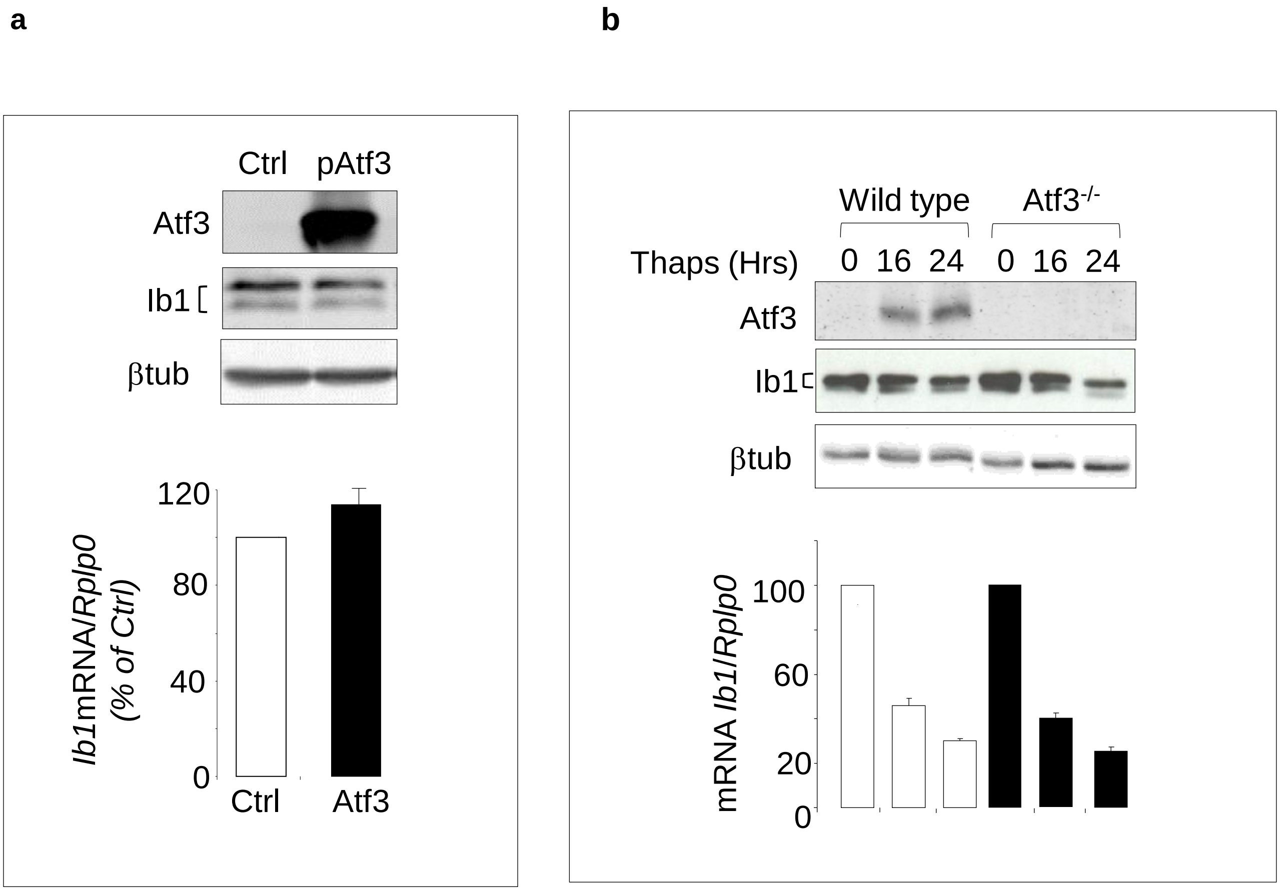

Supplement: Supplementary file 1 — Supplementary Figure 1: Role of Atf3 on the Ib1 protein and mRNA levels. [file 9158562.f1.pdf]
